# When orofacial pain needs a heart repair

**DOI:** 10.1002/cre2.359

**Published:** 2020-11-28

**Authors:** Daniela Adamo, Elena Calabria, Noemi Coppola, Giuseppe Pecoraro, Giuseppe Buono, Michele Davide Mignogna

**Affiliations:** ^1^ Department of Neurosciences, Reproductive and Odontostomatological Sciences University Federico II of Naples Naples Italy; ^2^ Department of Morphological and Functional Imaging, Haematology and Oncology Sciences University Federico II of Naples Naples Italy

**Keywords:** facial pain, homozygosis mutation for MTHFR, interatrial septum aneurysm, patent foramen ovale, right‐to‐left shunt

## Abstract

**Objectives:**

The association of chronic orofacial pain (COFP) and congenital heart disease has never previously been reported. We report the first case of COFP secondary to a right‐to‐left shunt (RLS) due to asymptomatic patent foramen ovale (PFO) in a patient with prothrombotic states.

**Materials and methods:**

A 48‐year‐old female patient presented with a 10‐month history of left‐sided facial pain who was initially diagnosed with persistent idiopathic facial pain (PIFP) on account of its similar characteristics. Magnetic resonance imaging (MRI) of the brain revealed gliosis and carotid siphon tortuosity; in addition, hyperhomocysteinaemia due to the homozygosis mutation for 5,10 MethyleneTetraHydroFolate Reductase was identified. Transcranial doppler ultrasonography was requested from a neurology consultant which revealed a high degree of RLS. Subsequently, a cardiological evaluation was performed; the specialist requested a transesophageal echocardiography that detected an interatrial septum aneurysm with PFO.

**Results:**

Based on the analysis of the patient's high degree of RLS, prothrombotic state and gliosis in relation to age, the cardiological consultant chose to perform a percutaneous closure of the PFO to avoid the risk of a cryptogenic stroke. After PFO closure, a complete remission of the pain was obtained.

**Conclusions:**

The disappearance of the pain supports the possible association between RLS and COFP. PFO with RLS has been suggested as a risk factor for cryptogenic stroke, especially in association with other thromboembolic risk factors. Therefore, the early detection, in this case, could be considered a possible lifesaver. Communication between different care providers is essential when the patient presents symptoms of facial pain which are of an atypical nature.

## 1 INTRODUCTION

Chronic orofacial pain (COFP) is an umbrella term used to describe several painful regional syndromes with a chronic, unremitting pattern causing suffering and disability in the affected population (Benoliel & Sharav, 2010). The differential diagnosis is complex and requires the analysis of the pattern of the pain, the investigation of associated diseases identified as primary and secondary forms of COFP and neurological examinations to identify any possible sensory deficit (ICOP, 2020).

The pain experience is fundamentally protective and is rarely present as a single symptom. Instead, it is often the first sign of a serious underlying condition in relation to different regions and aetiologies, which the clinician should be able to exclude through a careful clinical examination, and an analysis of the medical history of the patient, the pain characteristics and imaging (Vardeh et al., 2016). A multidisciplinary intervention by healthcare professionals is frequently essential in order to arrive at a proper diagnosis (Benoliel & Gaul, 2017).

Patent foramen ovale (PFO) is a remnant of a normal fetal anatomy that abnormally persists into adulthood common in approximately 30% of the general population. It is the most common anatomical cause of an interatrial right‐to‐left shunt (RLS). Patients with this congenital heart abnormality are usually asymptomatic because the defect is flap‐like and does not permit significant left‐to‐right shunting (Alakbarzade et al., 2020). However, some PFOs may open widely and provide a conduit for material such as thrombi, air or vasoactive peptides to travel from the venous to arterial circulation causing a paradoxical embolism. Therefore, several studies have implied that PFO with specific characteristics is associated with the onset of cryptogenic stroke, having no other identifiable cause. Significant factors include the size or height of the PFO, the degree of the RLS, and the presence of concomitant atrial septum aneurysm (Nakanishi et al., 2017).

For the majority of people, a PFO will remain undetected or appear only as a chance finding during cardiac investigation. Otherwise, the diagnosis is often made after the patient suffers a stroke, migraine‐like symptoms or decompression sickness while diving (Ioannidis & Mitsias, 2020).

New evidence suggests that PFO closure reduces the risk of recurrent ischaemic stroke in selected populations, such as those younger than 60 years of age with a high degree of RLS or an atrial septal aneurysm, particularly when a hypercoagulable state is found (Demulier et al., 2020; Nakanishi et al., 2017). Considerable evidence suggests that percutaneous PFO closure, in addition to antiplatelet therapy, can improve the treatment of migraine (Kumar et al., 2019).

The association between pain with different characteristics from migraine mimicking Persistent Idiopathic Facial Pain (PIFP) and a congenital heart abnormality has never previously been reported. We present the first case of COFP secondary to RLS due to asymptomatic PFO in a patient with prothrombotic states in which the PFO closure was associated with a complete remission of the pain.

## 2 MATERIAL AND METHODS

Written informed consent was given by the patient for the publication of this case report and the accompanying images previous an institutionally ethical approval.

### 2.1 Case presentation

A 48‐year‐old female patient was referred to the Oral Medicine Unit of the University Hospital of Federico II of Naples in October 2018 by her consultant neurologist on account of a 10‐month history of recalcitrant chronic pain affecting the left side of her face diagnosed as a PIFP. At the beginning, she had been unsuccessfully treated with amitriptyline (up to 50 mg/day) and pregabalin (up to 225 mg/day). Subsequently, sumatriptan (50 mg/daily) had been prescribed on suspicion of migraine without any results in terms of a remission of the pain. Finally, to exclude trigeminal autonomic cephalalgias, indomethacin (75 mg/daily) had been prescribed. None of these pharmacological treatments had served to resolve her symptoms and she had discontinued all these drugs due to the occurrence of side effects. She had undergone several investigations and no dental cause had been detected, although several tooth extractions had been performed without any relief.

At admission, the clinical and radiographic examination did not reveal any visible signs of any pathology. The pain was persistent, unilateral, continuous and commonly described as aching and dull, occurring daily with a moderate to severe intensity. It was poorly localized, not properly following the second branch of the left trigeminal nerve and diffuse to gingiva, teeth and ipsilateral jaw without head pain or facial sweating. Positive (hyperalgesia and allodynia) and negative (hypaesthesia and hypalgesia) somatosensory changes were not identified from qualitative and quantitative somatosensory testing. The pain was not associated with any paroxysms or any autonomic symptoms such as ipsilateral conjunctival injection, lacrimation, photophobia, nasal congestion, rhinorrhoea, nausea, vomiting or phonophobia. No trigger, exacerbating or relieving factors or neurological deficits were identified. There was no visible head or neck pathology and no cervical lymphadenopathy. Therefore, in line with her previous consultant and with ICOP clinical guidelines, a diagnosis of COFP resembling PIFP without somatosensory changes was made (ICOP, 2020).

**FIGURE 1:**
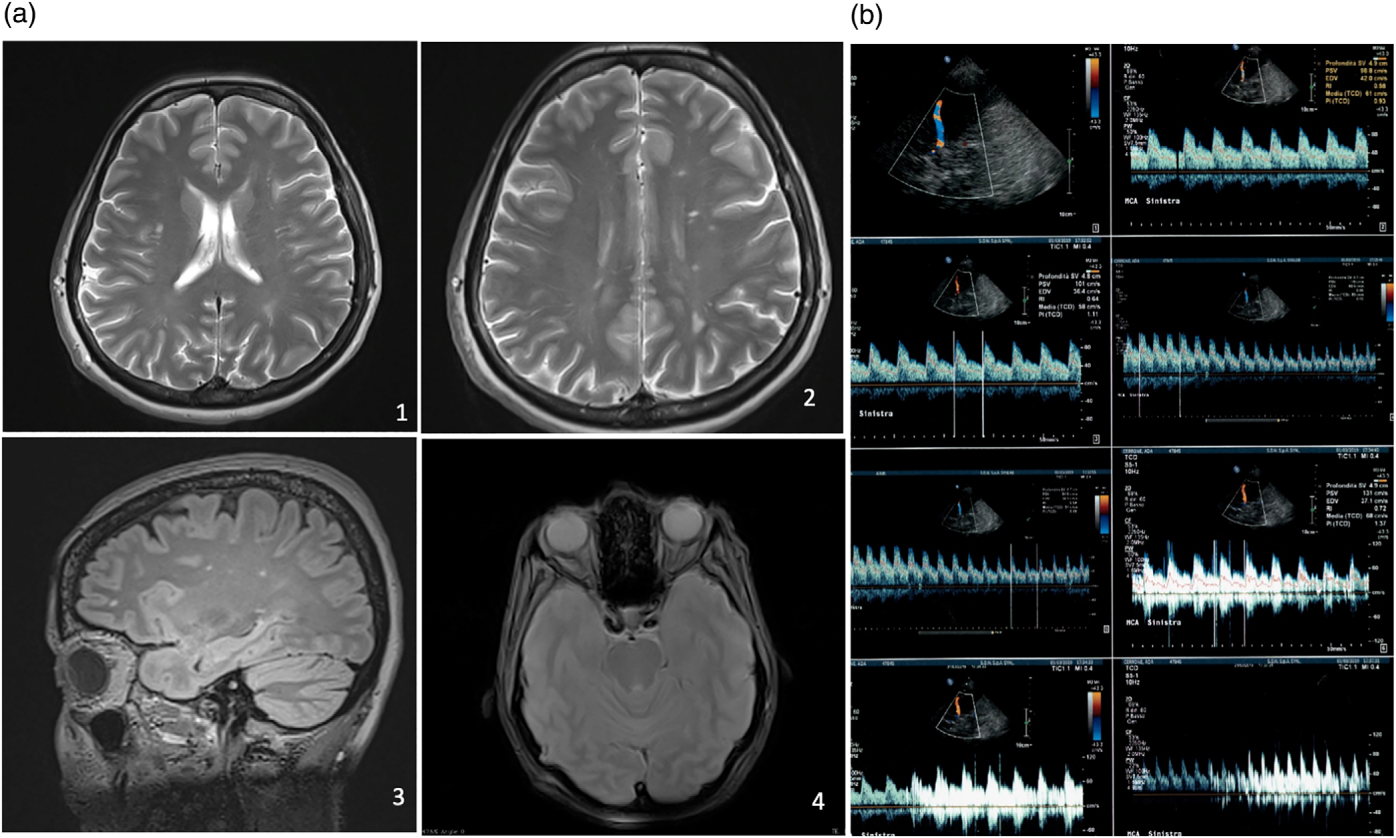
(a) Magnetic resonance imaging (MRI) of the brain and brainstem with and without intravenous paramagnetic contrast showed: (1)–(2) T2W TSE FSE TRA gliotic areas of the white substance of the brain; (3) 3D FLAIR SAG SAT gliotic areas in of the white substance of the brain. (4) GRADIENT AXIAL asymmetry and tortuosity of carotid siphons. (b) Transcranial Doppler Ultrasonography (TCD) with the agitated saline test showed a high degree of shunt with uncountable microembolic signals (the “curtain effect”)

She had a history of venous thromboembolism occurring at the age of 32 years and never evaluated. She did not report the presence of any other systemic diseases, or any history of depression, anxiety or psychiatric disorder. She was of normal weight (Body Mass Index: 20.5) and was not a smoker or drug‐taker. Routine blood tests, including folic acid, vitamin B12 and homocysteine level, were performed and hyperhomocysteinaemia (18 μmol/L) was identified. Therefore, a complete thrombophilic screening [Methylene TetraHydrofolate Reductase (MTHFR) C677T and 1298C mutations, Factor V mutation, Factor II mutation, lupus anticoagulant (LAC) panel, and protein C and S dosage] was requested, taking into account the thromboembolic risk to the patient. The 677C → T (A222V) mutation for MTHFR in homozygosity was identified. Magnetic Resonance Imaging (MRI) of the brain, brainstem and maxillo‐facial region with and without intravenous paramagnetic contrast was requested, highlighting gliosis and carotid siphon tortuosity. Therefore, neurological and cardiological consultations were requested. The multidisciplinary evaluation of the patient, who had been suffering for a long time from facial pain of an atypical nature, was essential to guide the request for the following instrumental examinations. Contrast‐enhanced Transcranial Doppler Ultrasonography (cTCD) with the agitated saline test was performed and showed a high degree of RLS (grade IV) with uncountable microembolic signals (the “curtain effect”). The shunt was classified as permanent because it was present under basal conditions without using the Valsalva maneuvre (Alakbarzade et al., 2020) (Figure 1a,b).

Subsequently, a Transesophageal Echocardiography (TEE) detected an interatrial septum aneurysm with PFO. Therefore, a diagnosis of PFO associated with interatrial septum aneurysm with a high degree of RLS in a patient with hypercoagulable states was made.

The association of two different risk factors for ischemic stroke in relation to the age of the patient guided the therapeutic plan of the consultant cardiologist. The patient underwent a percutaneous closure of the PFO with an Amplatzer PFO occluder 16 mm. Therapy with clopidogrel (75 mg/day) and acetylsalicylic acid (100 mg/day) was prescribed for 3 months followed by acetyl salicylic acid alone in association with folic acid supplementation. We evaluated the patient directly after 3 months from the PFO closure; in this follow‐up visit the patient reported a gradual improvement of her symptoms over the previous 3 months, and a complete remission of the pain at the time of consultation without any further treatments. After 6 months the patient continues to stay in well‐being and is currently being treated only with an antiaggregant and folic acid supplementation.

## 3 DISCUSSION

PFO accounts for 95% of all RLS. TEE and c‐TCD are the complementary imaging procedures of choice in adult patients with suspected PFO with RLS (Ioannidis & Mitsias, 2020; Nedeltchev & Mattle, 2006) or in patients at risk of cryptogenic stroke, and provide an indication for the distinction between “innocent” and “suspicious” shunts (Nedeltchev & Mattle, 2006).

PFO with RLS has been recognized as being strongly associated with other types of chronic facial pain, such as migraine, especially the type with aura (approximately 48%) (Kumar et al., 2019; Liu et al., 2018), with an improvement of the symptoms reported after the PFO closure (He et al., 2019).

The pathogenetic mechanisms of this type of orofacial pain mimicking PIFP can be hypothesized to be similar to that of migraine. Multiple mechanisms, poorly understood, could be considered to explain the possible causal relationship (Hajra & Bandyopadhyay, 2016).

First, the passage of blood directly from the right‐to‐left atrium (bypassing the normal filtering activity of the lungs) might allow that paradoxical emboli or microthrombi originating in the venous circulation to enter the systemic and cerebral circulation and so cause a transient ischemia and microinfarcts in the brain that may be a trigger for the onset of facial pain (Nakanishi et al., 2017). The presence of gliosis found at MRI of the brain in a young patient could indirectly correlate to thrombotic white matter changes, moreover, the hypercoagulability status could predispose to paradoxical emboli (Sachdev, 2005).

A second possibility is that certain metabolites, such as serotonin, nitric oxide, and the kinins, bypass the pulmonary circulation via the PFO, particularly in a larger PFO with a high degree of shunt, enter the systemic circulation and cause an irritation of the trigeminal nerve and brain vasculature producing pain (Liu et al., 2018). In particular, the increase in the level of serotonin could have a role. Normally serotonin is metabolized by the pulmonary monoamine oxidase enzyme but in the presence of a PFO, it is shunted away from the lungs with the increase in the level. In addition, serotonin is released from aggregating platelets and every condition that increases platelet activation or aggregation can increase the level of this metabolite. Probably, in our case the use of an antiplatelet drug contributed to alleviating pain through the reduction in the formation of platelet–fibrin complexes and serotonin level (Hajra & Bandyopadhyay, 2016).

Finally, the last hypothesis is that our patient presented a type of migraine without aura with atypical characteristics (He et al., 2019). Previous studies report cases of migraine with an atypical and unique location of pain in the lower two distributions of the trigeminal nerve without any autonomic symptoms, highlighting the possibility of a convergence mechanism in the transmission of pain involving the trigeminal nucleus caudalis and neural plasticity (Sathasivam & Sathasivam, 2013). In our case, there is some degree of doubt in relation to this hypothesis on account of the absence of any response to triptan medication.

Early detection and treatment is essential in PFO with RLS because this condition has been suggested as a risk factor for cryptogenic stroke in patients younger than 55 years through a mechanism probably consisting in a paradoxical embolism, particularly when a hypercoagulability status is associated. Over a fifth of patients with PFO presented at least one definite abnormality on a thrombophilia test (Ioannidis & Mitsias, 2020). Therefore, it seems reasonable to screen these patients, particularly those with a previous history of thromboembolic events (Rodriguez & Homma, 2003). In contrast, the introduction of this evaluation in a patient with facial pain remains controversial despite new evidence suggested an association between COFP and hyperhomocysteinaemia (Chiang et al., 2020).

In our case, the detection of two risk factors, namely age and a previous history of thromboembolic events in the patient, in association with PFO, persuaded the cardiologist to undertake a PFO closure procedure for the secondary prevention of paradoxical cerebral events.

The surprising disappearance of pain 3 months after the PFO closure, has suggested the hypothesis of a causal relationship between COFP and PFO with RLS, considering in addition the homozygous mutation of MTHFR and the fact that, until now, the patient is in treatment only with an antiaggregant and folic acid supplementation. Further studies and other case reports are needed to confirm our suggestion.

It must also be emphasized that the closure of a PFO is a not risk‐free. Surgical procedures to manage complications of transcatheter interventions may be needed in up to 8% of cases. Serious complications such as thrombus formation on the implant device, thromboembolism related to the implant device, cardiac perforation, infective endocarditis, and cardiac arrhythmias have been reported (Abaci et al., 2013).

## 4 CONCLUSIONS

To the best of our knowledge, this is the first case description of facial pain secondary to a RLS due to an asymptomatic PFO in a patient with a prothrombotic state where the disappearance of pain after the percutaneous closure of the PFO supports the possible association between a RLS and COFP suggesting a new type of secondary orofacial pain. However, the study leaves an unresolved question: namely, whether the association is coincidental or causal. To address this point, the mechanism of action has to be further elucidated.

The onset of facial pain can often mask organic conditions, and, in this context, in our patient the symptom could be considered lifesaving. This report shows that a careful evaluation of the clinical history, together with an interdisciplinary collaboration and a specific work up including neuroimaging studies, and the evaluation of hypercoagulability status is recommended in every case of facial pain in order both to achieve an earlier and more accurate recognition and treatment to avoid diagnostic delay, which could sometimes be fatal for the patient. PFO closure cannot be recommended as a routine treatment for COFP but only when other medical conditions could increase the risk of stroke due to the fact that the complications of this procedure may be serious and significant in comparison to the nonlife threatening nature of the pain.

## CONFLICTS OF INTEREST

The authors declare that there are no conflicts of interest regarding the publication of this article.

## AUTHOR CONTRIBUTIONS

All the authors have contributed to the work and are familiar with the primary data. Each author has read the final version of the manuscript and has approved its content. All the authors have agreed to have their name added to the paper. Drs. D. Adamo and G. Pecoraro designed the study, examined the patient and edited the paper; Dr. Buono performed the imaging; Drs. E. Calabria and N Coppola and Prof M. D. Mignogna drafted and edited the paper.

## CONSENT

Written informed consent was given by the patient for the publication of this case report and the accompanying images.

## Data Availability

The data that support the findings of this study are available from the corresponding author upon reasonable request.

## References

[cre2359-bib-0001] Abaci, A. , Unlu, S. , Alsancak, Y. , Kaya, U. , & Sezenoz, B. (2013). Short and long term complications of device closure of atrial septal defect and patent foramen ovale: Meta‐analysis of 28,142 patients from 203 studies. Catheterization and Cardiovascular Interventions, 82(7), 1123–1138. 10.1002/ccd.24875 23412921

[cre2359-bib-0002] Alakbarzade, V. , Keteepe‐Arachi, T. , Karsan, N. , Ray, R. , & Pereira, A. C. (2020). Patent foramen ovale. Practical Neurology, 20(3), 225–233. 10.1136/practneurol-2019-002450 32299831

[cre2359-bib-0003] Benoliel, R. , & Gaul, C. (2017). Persistent idiopathic facial pain. Cephalalgia, 37(7), 680–691. 28425324 10.1177/0333102417706349

[cre2359-bib-0004] Benoliel, R. , & Sharav, Y. (2010). Chronic orofacial pain. Current Pain and Headache Reports, 14(1), 33–40. 10.1007/s11916-009-0085-y 20425212

[cre2359-bib-0006] Chiang, M. L. , Chiang, C. P. , & Sun, A. (2020). Anemia, hematinic deficiencies, and gastric parietal cell antibody positivity in burning mouth syndrome patients with or without hyperhomocysteinemia. Journal of Dental Science, 15(2), 214–221. 10.1016/j.jds.2020.04.013 PMC7305457 32595904

[cre2359-bib-0007] Demulier L , Paelinck BP , Coomans I , Hemelsoet D , De Backer J , Campens L , & De Wolf D . (2020). A new dimension in patent foramen ovale size estimation. Echocardiography, 37, 1049–1055. 10.1111/echo.14696 32634292

[cre2359-bib-0008] Hajra, A. , & Bandyopadhyay, D. (2016). Patent foramen Ovale and migraine: Casual or causal. North American Journal of Medical Sciences, 8(3), 169–170. 10.4103/1947-2714.179139 27114976 PMC4821098

[cre2359-bib-0009] He, Y. D. , Yan, X. L. , Qin, C. , Zhang, P. , Guo, Z. N. , & Yang, Y. (2019). Transcatheter patent foramen Ovale closure is effective in alleviating migraine in a 5‐year follow‐up. Frontiers in Neurology, 10, 1224. 10.3389/fneur.2019.01224 31803135 PMC6877730

[cre2359-bib-0005] ICOP . (2020). International Classification of Orofacial Pain, 1st edition (ICOP). Cephalalgia, 40(2), 129–221. 10.1177/0333102419893823 32103673

[cre2359-bib-0010] Ioannidis, S. G. , & Mitsias, P. D. (2020). Patent foramen Ovale in cryptogenic ischemic stroke: Direct cause, risk factor, or incidental finding? Frontiers in Neurology, 11, 567. 10.3389/fneur.2020.00567 32670184 PMC7330017

[cre2359-bib-0011] Kumar, P. , Kijima, Y. , West, B. H. , & Tobis, J. M. (2019). The connection between patent foramen Ovale and migraine. Neuroimaging Clinics of North America, 29(2), 261–270. 30926116 10.1016/j.nic.2019.01.006

[cre2359-bib-0012] Liu, Y. , Li, S. , Wang, R. , Han, X. , Su, M. , Cao, X. , … Yu, S. (2018). A new perspective of migraine symptoms in patients with congenital heart defect. Headache, 58(10), 1601–1611. 30444273 10.1111/head.13453

[cre2359-bib-0013] Nakanishi, K. , Yoshiyama, M. , & Homma, S. (2017). Patent foramen ovale and cryptogenic stroke. Trends in Cardiovascular Medicine, 27(8), 575–581. 28709812 10.1016/j.tcm.2017.06.016

[cre2359-bib-0014] Nedeltchev, K. , & Mattle, H. P. (2006). Contrast‐enhanced transcranial Doppler ultrasound for diagnosis of patent foramen ovale. Frontiers of Neurology and Neuroscience, 21, 206–215. 10.1159/000092432 17290139

[cre2359-bib-0015] Rodriguez, C. J. , & Homma, S. (2003). Hypercoagulable states in patients with patent foramen ovale. Current Hematology Reports, 2(5), 435–441. 12932318

[cre2359-bib-0016] Sachdev, P. S. (2005). Homocysteinemia and brain atrophy. Progress in Neuro‐Psychopharmacology & Biological Psychiatry, 29(7), 1152–1161. 10.1016/j.pnpbp.2005.06.026 16102882

[cre2359-bib-0017] Sathasivam, S. , & Sathasivam, S. (2013). Patent foramen ovale and migraine: What is the relationship between the two? Journal of Cardiology, 61(4), 256–259. 10.1016/j.jjcc.2012.12.005 23484805

[cre2359-bib-0018] Vardeh, D. , Mannion, R. J. , & Woolf, C. J. (2016). Toward a mechanism‐based approach to pain diagnosis. The Journal of Pain, 17(9 Suppl), T50–T69. 10.1016/j.jpain.2016.03.001 27586831 PMC5012312

